# Treatment of Anomalous Coronary Arteries—Surgical Revascularisation Using the Pure Internal Thoracic Artery Technique

**DOI:** 10.3390/jcdd10040155

**Published:** 2023-04-02

**Authors:** Ramon L. James, Sudeep Das De, Sanjeet Singh Avtaar Singh, John Dreisbach, Stuart Watkins, Nawwar Al-Attar

**Affiliations:** 1 Department of Cardiac Surgery, Golden Jubilee National Hospital, Glasgow G81 4DH, UK; 2 Department of Radiology, Golden Jubilee National Hospital, Glasgow G81 4DH, UK; 3 Department of Cardiology, Golden Jubilee National Hospital, Glasgow G81 4DH, UK

**Keywords:** anomalous aortic origin of coronary artery (AAOCA), pure internal thoracic artery (PITA), coronary artery bypass grafting (CABG), congenital artery anomaly

## Abstract

OBJECTIVES: To evaluate the use of CABG utilising an isolated pedicled Right Internal Thoracic Artery (RITA) or Left Internal Thoracic Artery (LITA) or the Pure Internal Thoracic Artery (PITA) technique to treat anomalous aortic origin of coronary artery (AAOCA). METHODS: A retrospective review of all patients at our institution over an 8-year period (2013–2021) who underwent surgery for AAOCA was performed. Data assessed included patient demographics, initial presentation, morphology of coronary anomaly, surgical procedure, cross-clamp time, cardiopulmonary bypass time, and long-term outcome. RESULTS: A total of 14 patients underwent surgery, including 11 males (78.5%) with a median logistic EuroSCORE of 1.605 (IQR 1.34). The median age was 62.5 years (IQR 48.75). Presentation was angina (7 patients), acute coronary syndrome (5 patients), incidental findings in aortic valve pathology (2 patients). AAOCA morphology varied: RCA from left coronary sinus (6), RCA from left main stem (3), left coronary artery from the right coronary sinus (1), left main stem arising from right coronary sinus (2) and circumflex artery arising from the right coronary sinus (2). Overall, 7 patients had co-existing flow-limiting coronary artery disease. CABG was performed using either a pedicled skeletonized RITA, LITA or PITA technique. There was no perioperative mortality. Overall median follow-up time was 43 months. One patient presented with recurrent angina secondary to graft failure at 2 years and there were two non-cardiac-related deaths at 4 and 35 months. CONCLUSION: The use of internal thoracic artery grafts can provide a durable treatment option in patients with anomalous coronary arteries. The potential risk of graft failure in patients with no flow-limiting disease should be very carefully considered. However, a proposed benefit of this technique is the use of a pedicle flow to increase the long-term patency. More consistent results are obtained when ischaemia can be demonstrated preoperatively.

## 1. Introduction

Coronary artery anomalies are a rare, heterogenous group of congenital disorders of the coronary arterial circulation, comprising the anomalous origin of the coronary ostium, vessel course, and/or unusual number [1,2]. They are often discovered incidentally with a prevalence of approximately 0.17% in autopsy cases and 1.2% angiographically [1,3,4,5,6,7,8]. The occurrence of symptoms, including sudden cardiac death, is largely dependent on the anomalous aortic origin of the coronary artery (AAOCA), as well as the presence of a “malignant course”, particularly inter-arterial. It is the second leading cause of sudden cardiac death (SCD) in otherwise healthy young adults [9,10,11].

There remains controversy regarding the ideal management of these patients. Expert consensus guidelines have been developed regarding indications for intervention [12]. This includes medical management, such as watchful waiting with strenuous activity restriction, and various surgical therapeutic options. 

Current surgical therapeutic options include the unroofing of the coronary artery [13,14,15], coronary re-implantation [16,17], or patch arterioplasty/ostial reconstruction. Distal coronary artery bypass grafting (CABG) has been described as a possible treatment option; however, long term data regarding durability remain limited. Few authors have described early graft failure as secondary to competitive flow, particularly in younger patients receiving arterial conduits [18,19]. There is further suggestion that proximal ligation of the target vessel at the time of CABG may improve the long-term patency of the graft. There is limited research into the use of total arterial revascularization in treatment of this condition in patients with/without occlusive coronary artery disease. 

As a result, the aim of this study was to establish the use of coronary artery bypass grafting (CABG) using an isolated pedicled right internal thoracic artery (RITA) or left internal thoracic artery (LITA) or as a combination using the purely internal thoracic artery (PITA) technique to treat AAOCA. 

## 2. Results

**Figure 1:**
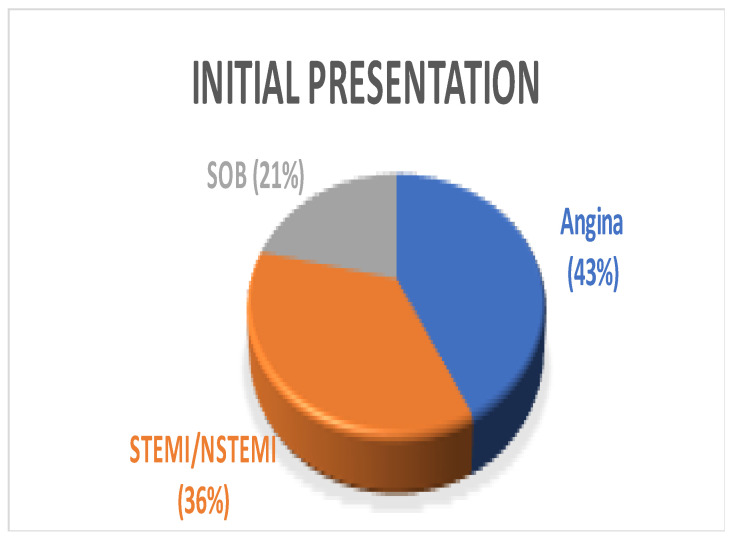
Pie Chart showing varying initial presentation among patients. SOB—shortness of breath, STEMI—ST segment elevation myocardial infarction, NSTEMI—Non-ST segment elevation myocardial infarction.

**Figure 2:**
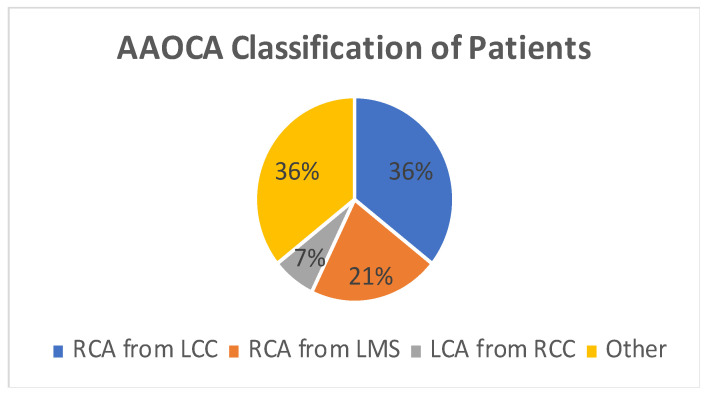
Pie Chart showing coronary artery morphology among patients. AAOCA—Anomalous aortic origin of coronary artery, RCA—right coronary artery, LCC—left coronary cusp, LMS—left main stem, LCA—left coronary artery, RCC—right coronary cusp.

**Figure 3:**
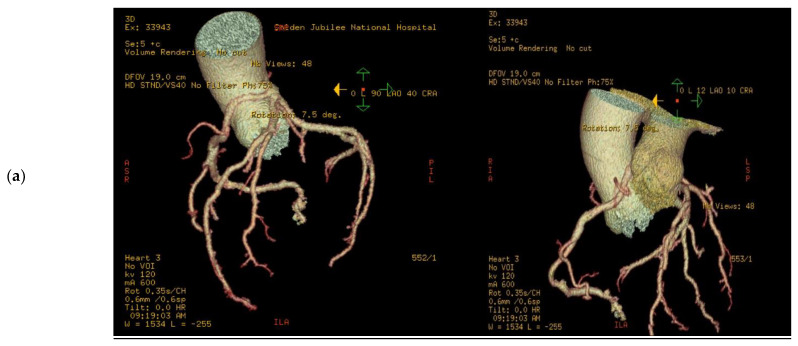

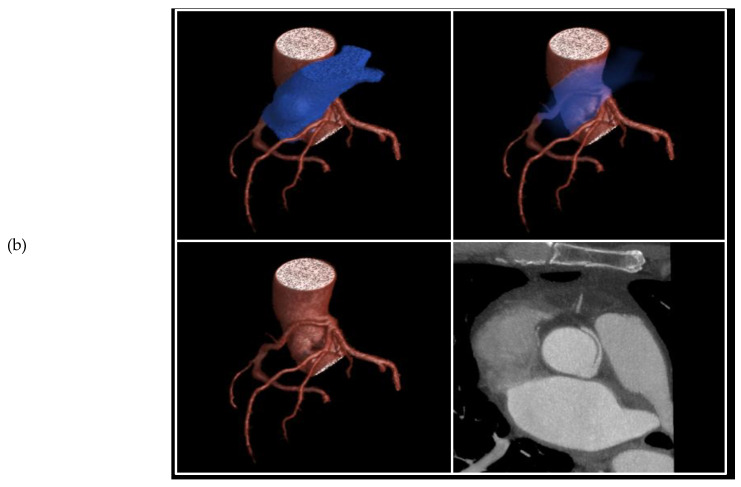
3D volume rendered images of CTCA in patient showing (**a**) RCA originating from LMS with inter-arterial course; (**b**) rendering with effacement of pulmonary artery and cross-sectional study.

A total of 14 patients underwent surgery for anomalous coronary arteries during the 8-year period. The median age was 62.5 years (IQR 48.75). There were 11 males (78.5%) with a median logistic EuroSCORE of 1.605 (IQR 1.34). The initial presentation of these patients included Angina (7 patients), Acute coronary syndrome (5 patients), and 2 patients with incidental findings of coronary artery anomalies while being investigated for aortic valve pathology (Figure 1). The morphology of the coronary artery anomalies varied (Figure 2) with the commonest being an anomalous right coronary artery (RCA) from the left coronary sinus (6 patients), followed by RCA from left main stem (3 patients) (Figure 3), left coronary artery from the right coronary sinus (1 patient), left main stem arising from right coronary sinus (2 patients), and circumflex artery arising from the right coronary sinus (2 patients). Overall, 7 patients had co-existing flow-limiting coronary artery disease.

A total of 10 patients underwent isolated CABG, 3 patients underwent concomitant aortic valve replacement (AVR), and 1 patient underwent concomitant AVR plus ascending aorta replacement. The median cross-clamp and cardiopulmonary bypass time were 59 and 74 minutes, respectively. CABG in all patients was performed using a pedicled skeletonized RITA, LITA or PITA technique to bypass the anomalous coronary artery, as well as treat any pre-existing coronary artery disease. There was no perioperative mortality. Overall, median follow-up time was 43 months. Particularly, one patient presented with recurrent angina secondary to graft failure at 2 years, and there were two non-cardiac related deaths at 4 and 35 months from ischaemic colitis and bowel obstruction due to complicated diverticular disease, respectively.

## 3. Discussion

AAOCA can be a cause of SCD, particularly in young patients participating in high-intensity exercise [20]. The ideal management of these patients remains uncertain. The following options have been advocated:

1. Non-Surgical: Exercise (high-intensity) restriction
2. Surgical:
  - Unroofing of coronary artery;
  - Patch arterioplasty;
  - Coronary re-implantation;
  - Coronary artery bypass grafting.

The most common anomalous coronary morphologies described in the literature in decreasing frequency are [21]:

1. Circumflex artery from the right sinus of Valsalva;
2. A single coronary artery from the left sinus of Valsalva;
3. Both coronary arteries from the right sinus of Valsalva;
4. Left anterior descending artery (LAD) from the right sinus of Valsalva;
5. Right coronary artery (RCA) arising from the left sinus of Valsalva;
6. Left main coronary artery arising from the right sinus of Valsalva.

Interestingly, this was not seen in our patient cohort. Rather, the right coronary artery from the left coronary sinus of Valsalva was the most common in 6/14 patients (42.8%). These patients tend to have an inter-arterial course (between aorta and pulmonary artery), often characterised as “malignant”. Anomalous coronary arteries arising from the opposite sinus of Valsalva (SoV) are the rarest morphology but are of particular interest due to the probable higher risk of myocardial ischaemia and SCD, especially in young patients partaking in strenuous physical activities. Prevalence is estimated at 0.26% in the general population (0.03% for left coronary ACAOS; L-ACAOS, 0.23% for right coronary ACAOS; R-ACAOS) [22,23]. The inter-arterial course itself is unlikely to be the predominant factor resulting in ischaemia, but rather a marker of several high-risk anatomical features. These features include, slit-like ostium, acute take-off angle, proximal narrowing with elliptical vessel shape and an intramural course. The intramural component is the most likely factor to predispose patients to myocardial ischaemia as its course is within the tunica media of the aortic wall and is likely to be compressed during systole. This is more probable than the initial postulated occlusion of the anomalous coronary artery by the extrinsic compression from a low-pressured pulmonary artery. Additionally, at the site of closest aortopulmonary proximity, the anomalous segment would run within the aortic wall [11,24,25]. 

The indications to intervene are controversial as it is generally acceptable to offer intervention to patients with symptoms. These symptoms can include syncope associated with documented or reasonably suspected ventricular arrhythmia, high-risk ventricular arrhythmias, chest pain consistent with angina, aborted SCD or cardiac arrest, and evidence of ischaemia on provocative testing [26,27]. All 14 patients were symptomatic with angina (7 patients), acute coronary syndrome (5 patients), and 2 patients with incidental findings of coronary artery anomalies while being investigated for symptomatic aortic valve pathology. 

However, the 2008 ACC/AHA guidelines also give recommendations for intervention in asymptomatic patients. The anomaly with the highest predictor of SCD is an inter-arterial AAOLCA with class I indications (27). A patient with AAOCA who does not meet the criteria for surgical intervention should only make a final decision for participation in competitive or high-intensity recreational sports after a detailed family discussion and counselling regarding the risks and benefits of observation vs. surgical intervention.

The role of CABG in patients without obstructive coronary artery disease remains undetermined, given the potential for competitive flow from native vessels resulting in early graft failure [22]. However, the long-term graft patency rate of the internal thoracic artery is superior to the saphenous vein graft, and as a result, some authors have advocated for its use in CABG in this subset of patients [28]. Sabik and colleagues proposed that proximal ligation of the anomalous coronary target is performed at the time of coronary bypass to decrease the risk of early graft failure, particularly in those patients receiving arterial grafts in the absence of occlusive native coronary artery disease [29]. However, ligation of the RCA with minimal or no occlusive disease is of concern due to the inability of the ITA graft to compensate for acute ligation of a patent vessel, which can result in hypoperfusion, ischaemia, and increased mortality [30]. It also presents a moral dilemma of occluding a “normal” vessel. As a result, CABG may be a better treatment option in patients with established coronary artery disease and documented ischaemia, where it shows good early and midterm results [31,32]. 

All 14 patients were treated with total arterial revascularization using only the internal thoracic artery harvested in a skeletonised fashion. Configurations varied as follows: Isolated RITA—RCA (5 patients), Isolated LITA—LAD (1 patient), Y- configurations (in situ LITA and free RITA). This is the first report of treatment of this condition with the PITA technique. In our patient cohort, 7 patients (50%) had co-existing occlusive coronary artery disease. 

Specifically, one patient presented with recurrent angina secondary to graft failure at 2 years follow-up confirmed on coronary angiography. He was a 57 yr/M with absence of flow-limiting disease pre-operatively with his coronary artery morphology being RCA from left main stem (LMS). Ischaemia has not been demonstrated by perfusion techniques or exercise testing. He refused further re-intervention and is managed medically.

Fedoruk et al. [18] described 40% late graft occlusion in 5 patients with AAORCA treated with right internal mammary artery graft, while Tavaf- Motamen et al. [19] reported 2 patients treated with CABG for AAORCA, both of whom had early graft failure with recurrence of symptoms and graft failure. As a result, they proposed the proximal ligation of the target vessel in patients with absent occlusive disease. Based on our experience, the graft failure rate was more favourable at 7% (1/14 patients).

## 4. Methods

A retrospective review of all patients at our institution over an 8-year period (2013–2021) who underwent surgery for AAOCA was performed. Permission from the local clinical governance board was obtained and assigned coding 1520. Given the retrospective nature of the study, informed patient consent was waived. Data assessed included patient demographics, initial presentation, morphology of coronary anomaly, EuroSCORE, surgical procedure, cross-clamp time, cardiopulmonary bypass time and long-term outcome. All patients were discussed at the coronary multidisciplinary meeting, and the consensus was discussed in shared decision-making with the patient and their next of kin.

## 5. Conclusions

The use of internal thoracic artery grafts can provide a durable treatment option in patients with anomalous coronary arteries. The potential risk of graft failure in patients with no flow-limiting disease should be very carefully considered. However, a proposed benefit of this technique is the use of a pedicle flow to increase long-term patency. More consistent results are obtained when ischaemia can be demonstrated pre-operatively. Proximal ligation of the coronary target in the absence of flow-limiting disease to reduce graft failure remains debatable.

## Data Availability

Not applicable.

## Abbreviations

CABG	Coronary artery bypass grafting
AAOCA	Anomalous aortic origin of coronary artery
SCD	Sudden Cardiac Death
RITA	Right internal thoracic artery
LITA	Left internal thoracic artery
PITA	Pure internal thoracic artery
IQR	Interquartile range
RCA	Right coronary artery
LMS	Left main stem
LCA	Left coronary artery
RCS	Right coronary sinus
LCS	Left coronary sinus
AVR	Aortic valve replacement
SoV	Sinus of Valsalva
ACC/AHA	American College of Cardiology/American Heart Association
AAOLCA	Anomalous aortic origin of left coronary artery
ACAOS	Anomalous coronary arteries with the origin of the anomalous vessel from the opposite sinus of Valsalva
LAD	Left anterior descending artery
ITA	Internal thoracic artery
Cx	Circumflex artery
OM1	1st Obtuse marginal artery
PDA	Posterior descending artery
m-AVR	Mechanical aortic valve replacement
t-AVR	Tissue/bioprosthetic aortic valve replacement
